# The Thermodynamic Flow-Force Interpretation of Root Nutrient Uptake Kinetics: A Powerful Formalism for Agronomic and Phytoplanktonic Models

**DOI:** 10.3389/fphys.2016.00243

**Published:** 2016-06-27

**Authors:** Erwan Le Deunff, Pierre-Henri Tournier, Philippe Malagoli

**Affiliations:** ^1^Université de Caen Basse-Normandie, UFR des Sciences, UMR EVACaen, France; ^2^Institut National de la Recherche Agronomique, UMR 950, Écophysiologie Végétale and Agronomie Nutritions NCSCaen, France; ^3^Laboratoire Jacques-Louis Lions, INRIA Paris, EPC Alpines and Université Pierre et Marie Curie Paris 06, UMR 7598Paris, France; ^4^Université Clermont Auvergne, Université Blaise Pascal, UMR 547, PIAFClermont-Ferrand, France; ^5^Institut National de la Recherche Agronomique, UMR 547 PIAFClermont-Ferrand, France

**Keywords:** ion transport modeling, influx, efflux, enzyme-substrate modeling, flow-force modeling, nitrate, potassium, phytoplankton

## Abstract

The ion influx isotherms obtained by measuring unidirectional influx across root membranes with radioactive or stable tracers are mostly interpreted by *enzyme-substrate-like* modeling. However, recent analyses from ion transporter mutants clearly demonstrate the inadequacy of the conventional interpretation of ion isotherms. Many genetically distinct carriers are involved in the root catalytic function. Parameters *Vmax* and *Km* deduced from this interpretation cannot therefore be regarded as microscopic parameters of a single transporter, but are instead macroscopic parameters (*V*${}_{m}^{app}$ and *K*${}_{m}^{app}$, apparent maximum velocity and affinity constant) that depend on weighted activities of multiple transporters along the root. The *flow-force* interpretation based on the thermodynamic principle of irreversible processes is an alternative macroscopic modeling approach for ion influx isotherms in which macroscopic parameters L_j_ (overall conductance of the root system for the substrate j) and π_j_ (thermodynamic parameter when J_j_ = 0) have a straightforward meaning with respect to the biological sample studied. They characterize the efficiency of the entire root catalytic structure without deducing molecular characteristics. Here we present the basic principles of this theory and how its use can be tested and improved by changing root pre- and post-wash procedures before influx measurements in order to come as close as possible to equilibrium conditions. In addition, the constant values of *Vm* and *Km* in the Michaelis-Menten (MM) formalism of *enzyme-substrate* interpretation do not reflect variations in response to temperature, nutrient status or nutrient regimes. The linear formalism of the *flow-force* approach, which integrates temperature effect on nutrient uptake, could usefully replace MM formalism in the 1-3-dimension models of plants and phytoplankton. This formalism offers a simplification of parametrization to help find more realistic analytical expressions and numerical solution for root nutrient uptake.

## Introduction

The kinetic patterns of ion uptake rates across roots, called ion influx isotherms, were first established in the 1960s by the pioneer work of Emanuel Epstein with ^86^Rb or ^42^K radioactive tracers for potassium uptake in barley (Epstein et al., 1963). These ion influx isotherms were later extended to other ions with radioactive or stable isotope tracers such as ^13^N and ^15^ N for nitrate, ^32^PO${}_{4}^{2-}$ and ^33^PO${}_{4}^{2-}$ for phosphate, and ^35^SO${}_{4}^{2-}$ and ^34^SO${}_{4}^{2-}$ for sulfate (Bieleski, 1973; Kochian et al., 1985; Lee and Drew, 1986; Siddiqi et al., 1989, 1990; Faure-Rabasse et al., 2002). The conventional *enzyme-substrate* interpretation of influx isotherms by Epstein's group refers to a dual mechanism of ion transport and defines two distinct transport systems: a high-affinity transport system (HATS) and a low-affinity transport system (LATS). HATS is characterized by a saturable kinetic pattern in the low ion concentration range (< 1 mM; Lee and Drew, 1986; Hole et al., 1990; Siddiqi et al., 1990; Aslam et al., 1992), whereas LATS exhibits saturable or linear behavior in the high ion concentration range (>1 mM; Pace and McClure, 1986; Siddiqi et al., 1990; Aslam et al., 1992; Kronzucker et al., 1995a).

The concept of transport systems (kinetic components of ion fluxes across the roots) deduced from the *enzyme-substrate* interpretation of influx isotherms is strengthened by the mathematical deduction of microscopic parameters such as *Vmax* and *Km* for the HATS and sometimes LATS, but shows its weakness in the case of the LATS mechanism when no enzymatic parameter can be set when its behavior is linear (Peuke and Kaiser, 1996). Although ion influx isotherms have been intensively used to validate molecular characterization of ion transporters in mutant analyses, recent analyses of ion transporter mutants for nitrate and potassium clearly demonstrate that the conventional *enzyme-substrate* interpretation is inadequate (Cerezo et al., 2001; Filleur et al., 2001; Li et al., 2007; Britto and Kronzucker, 2008; Alemán et al., 2011). Many carriers provided by genetically distinct gene families are involved in the root catalytic function (Touraine et al., 2001; Britto and Kronzucker, 2008; Alemán et al., 2011), and some transporters show double affinity depending on their phosphorylation status, as observed for the NRT1.1 (renamed NPF6.3) nitrate transporter (Liu and Tsay, 2003; Ho et al., 2009). *Vmax* and *Km* parameters deduced from an *enzyme-substrate* interpretation cannot therefore be regarded as microscopic parameters of a single transporter, but are instead macroscopic parameters (*Vmapp* and *Kmapp*) that reflect the sum of single activities of multiple transporters along the root (Neame and Richards, 1972).

Histochemical GUS (β-glucuronidase) or GFP (Green Fluorescent Protein) activities of *pNRT::GUS* or *pNRT::GFP* in transgenic Arabidopsis plants has revealed that these carriers are located on the different membrane cell layers within the mature root, and can be arranged in series or parallel to form a complex catalytic structure (Guo et al., 2001, 2002; Girin et al., 2007). The concept of transport systems deduced from the interpretation of influx isotherms cannot therefore be merged or confounded with ion transporters because influx components correspond to subsumed activities of multiple transporters along the root (Le Deunff and Malagoli, 2014a,b). Likewise, the copy number of the genes is also increased by endoreduplication in root cells during their elongation (Hayashi et al., 2013) and by a genome redundancy in polyploid crop species such as oilseed rape and wheat. Both situations probably lead to an underestimation of the number of nitrate transporters, hampering the *enzyme-substrate* interpretation of nitrate uptake isotherms. It is also well demonstrated that ion influx is uneven along the roots (Lazof et al., 1992; Reidenbach and Horst, 1997; Colmer and Bloom, 1998; Sorgona et al., 2011).

Conventional measurements of influx rate across the root in kinetic patterns are most often made in transient conditions far removed from equilibrium, because emphasis is laid on unidirectional influx rate across the plasma membrane instead of net flux (Britto and Kronzucker, 2001a,b; Britto and Kronzucker, 2003a; Glass et al., 2002). The pre- and post-wash conditions used for measurements therefore induced thermodynamic perturbations of the root membranes (Britto and Kronzucker, 2001a,b; Szczerba et al., 2006). Thus as shown by Kronzucker and co-workers, the pre- and post-wash conditions used in unidirectional influx measurements exhibit minor discrepancies in the HATS range, but large discrepancies in the LATS range of nutrient ion concentrations (Britto et al., 2006; Szczerba et al., 2006). In alternative approaches such as *flow-force* or compartmental analysis by the tracer efflux method (CATE), the measurements of net influx or efflux rates are more accurate and less chaotic because they are performed in steady-state conditions and are close to equilibrium (Britto et al., 2006). These experimental approaches offer major opportunities to find new solutions to improve formalisms of ion uptake in agronomic models for agricultural purposes.

In this review, we discuss experimental procedures to measure ion influx across the root, and present the basic principles of *flow-force* theory established in the 1970s (Thellier, 1969, 1970a,b; Thellier et al., 1971a,b), how this theory has evolved (Thellier, 1973, 2012; Thellier et al., 2009) and how and why its formalism could be used in agronomic and phytoplankton models of nutrient ion uptake.

## Experimental protocols for enzyme-like vs. flow-force modeling

Although the effects of local ion status and/or uptake-wash regime on uptake isotherm kinetics have long been recognized as very important factors influencing kinetic responses (Cram and Laties, 1971; Leigh et al., 1973; Ayadi et al., 1974; Tinker and Nye, 2000), they have been discussed only in the recent literature (Britto and Kronzucker, 2001a, 2006; Szczerba et al., 2006). Here we show that experimental procedures used to measure unidirectional ion influx across root membranes to establish ion influx isotherms will be different according to the modeling type chosen: *enzyme-like* or *flow-force*.

### Influx rate measurements according to enzyme-like interpretation of root ion uptake

**Figure 1:**
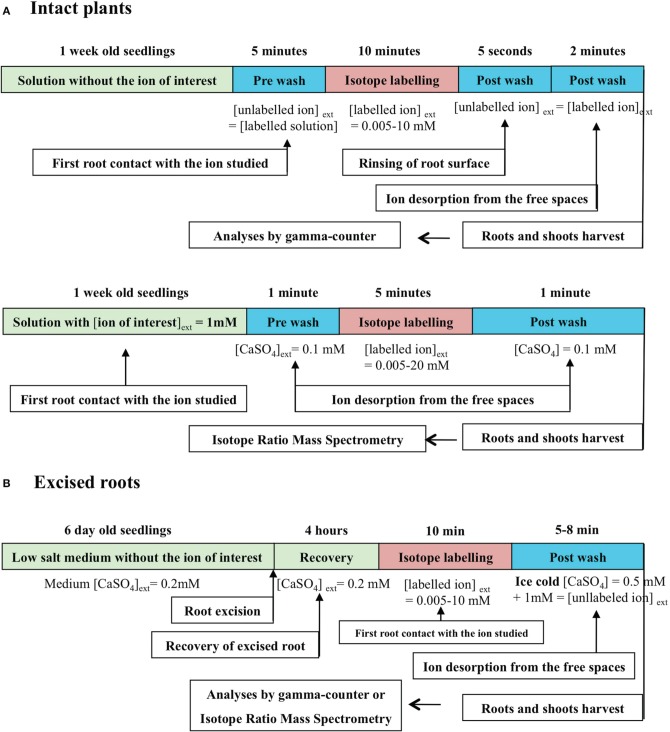
**Conventional procedure of ion influx measurements with radioactive or stable ion isotopes by stepwise increase of ion concentrations. (A)** Stepwise protocol for nitrate influx isotherms determination in intact plant roots of barley (from Siddiqi et al., 1989, 1990), spruce (Kronzucker et al., 1995a), and Arabidopsis (Filleur et al., 2001). **(B)** Stepwise protocol for K^+^ influx isotherms determination of excised roots of 6-day-old dark·grown maize seedlings (from Kochian and Lucas, 1982; Kochian et al., 1985).

In the conventional *enzyme-like* procedure, ion flux measurements across the root membranes at a given temperature (isotherm condition) are made on roots of intact plants (Polley and Hopkins, 1979; Siddiqi et al., 1989, 1990; Delhon et al., 1995; Faure-Rabasse et al., 2002) or excised roots (Epstein et al., 1963; Leigh et al., 1973; Kochian and Lucas, 1982; Kochian et al., 1985). The flux measurements with radioactive or stable tracers of the major nutrient ions present in soil (NO${}_{3}^{-}$, NH${}_{4}^{+}$, K^+^, PO${}_{4}^{2-}$) are performed over a short period of time: 5–10 min (Figure 1), because the half-life of the ion cytoplasmic pool is only 2–7 min (Presland and MacNaughton, 1984; Lee and Clarkson, 1986; Devienne et al., 1994; Muller et al., 1995). It is assumed that this short measurement time allows the assessment of influx from carriers located in the plasma membrane of epidermis root cell layer instead of net flux resulting from the difference between influx and efflux (Walker and Pitman, 1976). It is then critical to accurately determine the time needed to measure unidirectional ion influx, together with the durations of pre- and post-wash to equilibrate the apparent free spaces of the cell wall. As a rule, these durations are deduced from the half-life of tracer exchange between the apoplast and cytosol compartments, obtained by desorption experiments (Presland and MacNaughton, 1984; Lee and Clarkson, 1986; Devienne et al., 1994; Muller et al., 1995; Kronzucker et al., 1995b,c,d).

#### Pre- and post-wash steps during unidirectional measurement of influx rate with ion tracers in non-steady-state or non-equilibrium conditions

The determination of ion influx rate from plants never exposed to the ion of interest except for a 5 min pre-wash solution prior to influx measurement, or directly in labeling solution does not correspond to stationary or equilibrium conditions (Figure 1). Unidirectional influx rate values are obtained in a transient state although plants have the same nutrient status because they have been uniformly pre-treated (Figure 1). By contrast, the steady-state conditions can be defined as a situation in which ion fluxes in and out of the root cells of a substrate S_*j*_ do not fluctuate under given environmental conditions. In this stationary condition, the root system is crossed by a flow of matter or energy but the system properties do not change over time. In addition, the steady-state conditions do not rule out an active transport across the membrane that prevents many diffusive fluxes from ever reaching equilibrium. Such a situation is encountered in short-term isotopic labeling experiments in which the plant growth rate and nutrient solution are held constant (Britto and Kronzucker, 2001b, 2003a; Malagoli et al., 2008). The equilibrium is defined as no further net movement of solute in the lack of driving forces such as difference in concentration or electric field. In conventional pre-wash procedures presented in Figure 1A, the conditions for the ion of interest are far removed from the equilibrium or steady state conditions because plants are never exposed to this ion before influx measurements (Siddiqi et al., 1989, 1990; Tinker and Nye, 2000). These situations can be qualified as transient conditions because the system properties change over time. Likewise, during the post-wash step a low temperature is sometime applied to block the activity of influx and efflux carriers (Figure 1B). However, this condition may induce strong disturbances in measurements of ion influx from a thermodynamic point of view by modifying influx and efflux velocity characteristics of ion transporters (Britto and Kronzucker, 2001a).

#### Duration of pre- and post-wash steps is determined by compartmental analysis by tracer efflux

**Figure 2:**
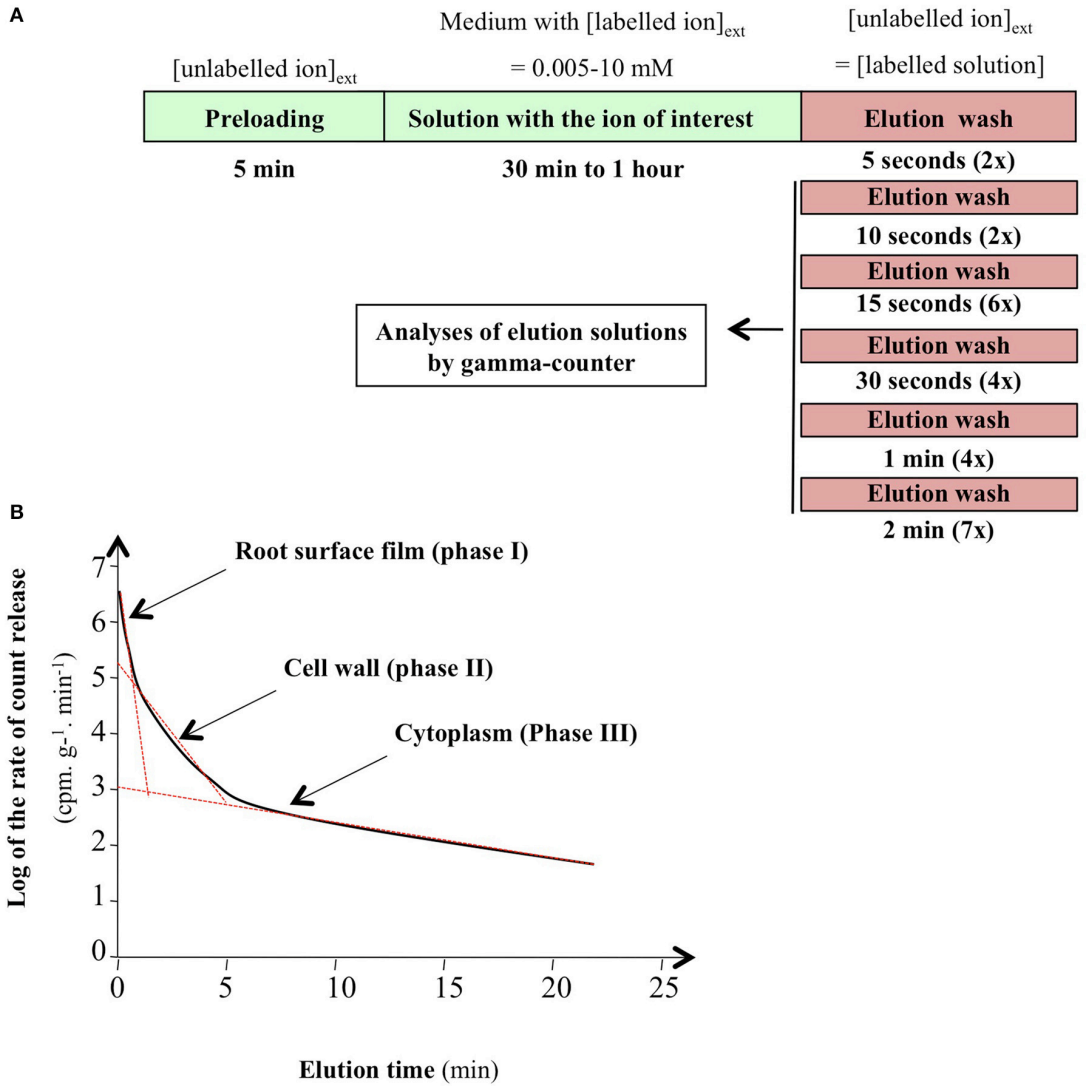
**Conventional procedure of root efflux analysis. (A)** Stepwise protocol for efflux in intact plant roots of spruce, barley, and rice. **(B)** Representative plot of ion efflux from roots of intact plants. Linear regression on semi-logarithmic plots was used to resolve phase I, II, and III corresponding to ion root surface film, cell wall, and cytosolic pools (from Clarkson, 1974; Kronzucker et al., 1995b; Britto and Kronzucker, 2003b).

Turnover in the tracer cytosolic pool is calculated from compartmental analysis by tracer efflux (CATE) from plants growing under steady-state conditions (Rauser, 1987; Cram, 1988; Siddiqi et al., 1991; Kronzucker et al., 1995b,c,d). Depending on the ion studied, the plant roots were exposed to a radioactive or stable tracer for 30 min to 1 h allowing both substantial labeling of the cytosolic pool and limited labeling of the vacuolar compartment under steady-state conditions. The plants were then transferred to a non-labeling solution of the same concentration, and a kinetic study of tracer elution due to its efflux was performed to monitor desorption from extra-cellular compartments, and then ion efflux from cytosol to external medium (Figure 2A). It is well-established that compartmental analysis from a semi-logarithmic plot of the time-course of ^13^N radiotracer efflux shows three different phases (Figure 2B). The successive phases are linked to the surface liquid film (phase I), cell wall composed of the water free space (WFS) and Donan free space (DFS; phase II) and cytosolic pool (phase III; Rauser, 1987; Kronzucker et al., 1995b,c,d; Britto and Kronzucker, 2003b). From these experiments, duration of ion tracer desorption (i.e., ion exchange between labeled and unlabeled ion in the apoplast) by washing with unlabeled nutrient solution is easily determined by the duration of phases I and II for different ion species (Siddiqi et al., 1989, 1990; Kronzucker et al., 1995b,c,d; Malagoli et al., 2008). The idea is to maximize ion removal from WFS and DFS while minimizing ion loss from the cytosolic pool. However, even in the steady-state conditions used, Kronzucker and co-workers have shown that elution of ^42^K tracer by washing of barley roots causes disturbance of ion efflux and leads to less accurate measurements of kinetic parameters. Accordingly, a new procedure involving continuous monitoring of bathing solution by removal and replacement of external solution aliquots was defined to improve the estimation of the kinetic constant, called sub-sample CATE (SCATE; Britto et al., 2006).

### Alternative flow-force procedure

**Figure 3:**
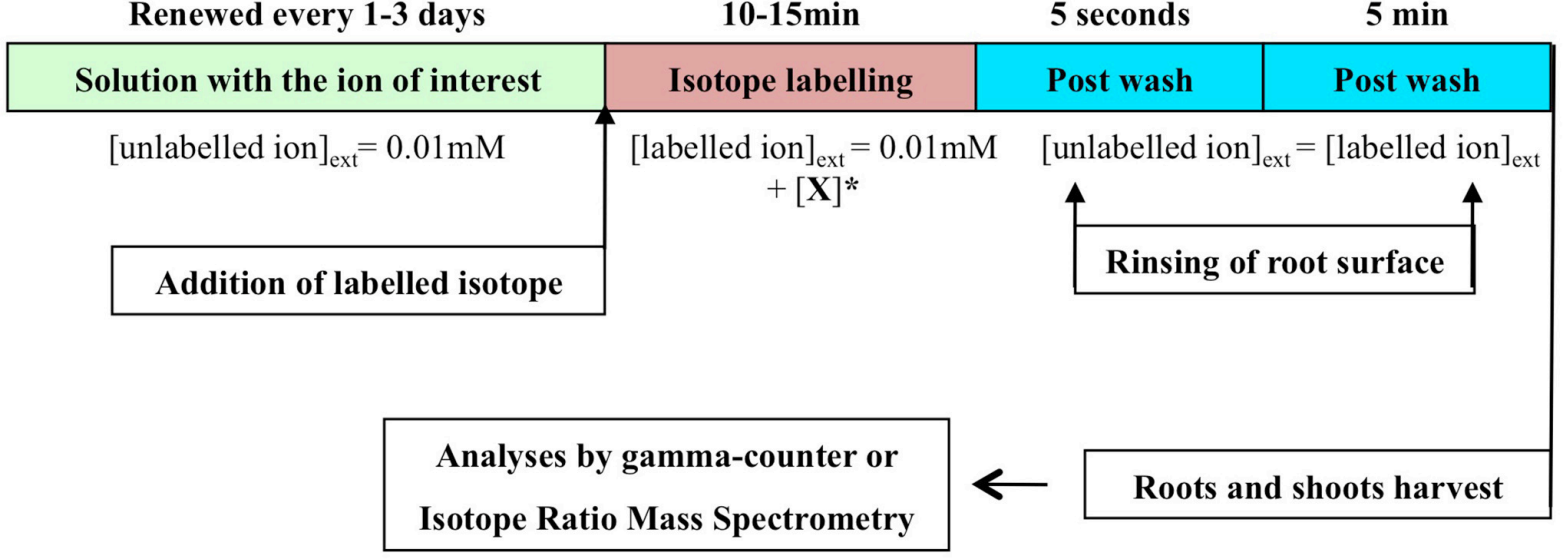
**Optimal experimental design for the *Flow-Force* modeling**. Stepwise protocol for influx in intact plant roots placed in conditions of steady-state growth. The stepwise increase of labeled ion concentration. = +[X] represents the concentrations of labeled ion needed to reach a desired final concentration for stepwise influx measurements.

In the *flow-force* procedure, the main difference is that flux measurements are performed on the roots at or close to equilibrium with the external nutrient solution (Ayadi et al., 1974; Tinker and Nye, 2000). The equilibrium is defined as no further net movement of solute in the absence of driving forces such as difference in concentration, or electric field. Accordingly, the plant roots placed in a non-labeled solution at a given external concentration were not washed before the tracer flux measurements to avoid destroying the initial state of equilibrium (Figure 3). The external concentration was smoothly increased by adding aliquots with labeled nutrient ion at a higher concentration, and the net flux was measured. The plants were then transferred to non-labeling solution at the same concentration to remove tracer from the cell wall. To some extent, the steady-state conditions used in SCATE procedure are close to that which should be followed in the flow-force analysis (Britto et al., 2006).

### Does the broad range of applied external concentrations have any biological significance in building isotherms?

For a long time in the *enzyme-like* conventional procedure, the maximal external ion concentrations used to build the isotherms exceeded those measured in non-anthropized or agricultural soil solutions by one or two orders of magnitude. For example, nutrient solution concentrations used in laboratory studies lay in the range 1 μM to 250 mM for nitrate (Siddiqi et al., 1989, 1990; Kronzucker et al., 1995a,b; Hu et al., 2009), nitrate concentrations being lower than 1 mM in non-anthropized soils and lower or equivalent to 10–20 mM after fertilization in agricultural soils (Reisenauer, 1966; NaNagara et al., 1976; Wolt, 1994; Britto and Kronzucker, 2005; Miller et al., 2007). Likewise, for potassium, the concentrations explored ranged from 1 μM to 10 or 100 mM (Epstein et al., 1963; Polley and Hopkins, 1979; Kochian and Lucas, 1982), typical K^+^ concentration in the soil solution ranging only from 1 μM to 6 mM (Maathuis, 2009). Furthermore, in the conventional *enzyme-substrate* wash procedure, measurement errors in unidirectional influx rate are less significant (10%) in a low range of ion concentration. However, the wash procedure induces errors of at least 30% in the high range of ion concentrations (Britto et al., 2006). Likewise, in the high range of concentrations for six major nutrient ions (Cl^−^, NO${}_{3}^{-}$, SO${}_{4}^{2-}$, K^+^, NH${}_{4}^{+}$, Na^+^), the efflux component increases, and efflux:influx ratios tend toward a value close to 1. Because the anion (A^−^) influx is mediated by an electrogenic symport mechanism with a general A^−^/2H^+^ stoichiometry, this result suggests that H^+^-ATPase pumps must run twice to counterbalance the anion efflux. Under a broad range of concentrations, this futile ion cycling probably has a large energy cost (Britto and Kronzucker, 2006). Taken all together, these results show that over a high range of ion nutrient concentrations, besides the lack of biological meaning of ion concentrations used, the kinetic patterns of the isotherms cannot be regarded as being accurate measurements of the unidirectional influx owing to the magnitude of the efflux component (Britto and Kronzucker, 2006).

## Enzyme-like modeling

**Figure 4:**
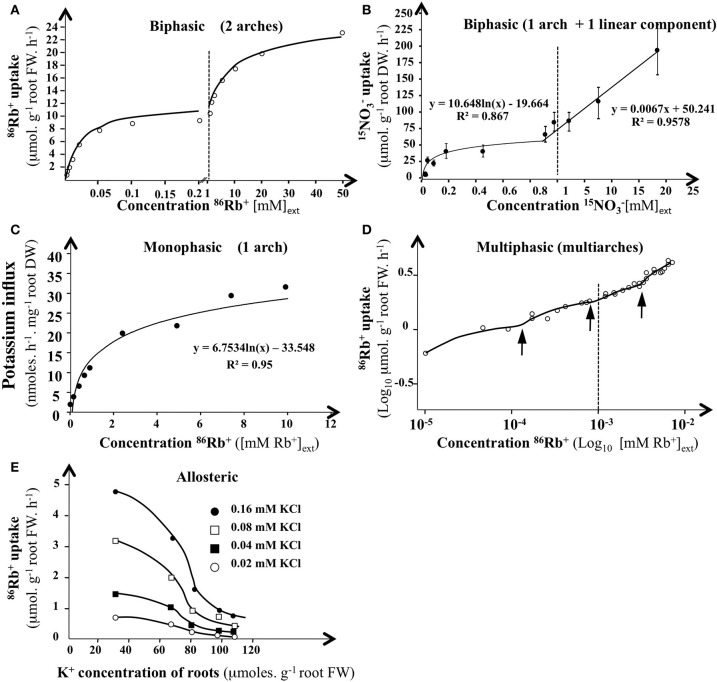
**Influx isotherms for K^+^ and NO${}_{3}^{-}$ absorption by plant roots in *H*. *vulgare* and *Arabidopsis*. (A)** Isotherms for ^42^K^+^ uptake of excised roots from 5-day·old dark-grown barley seedlings. K^+^influx rate was measured after 10 min of labeling at 30°C with a nutrient solution of 0.5 mM CaCI_2_ containing between 0.002 and 50 mM ^15^NO${}_{3}-$ (adapted from Epstein et al., 1963). **(B)** Isotherms for ^15^NO${}_{3}-$ of intact roots from 6-weeks-old *Arabidopsi*s plants. No${}_{3}-$influx rate was measured after 5 min of labeling at 25°C with a complete nutrient solution containing between 0.005 and 20 mM ^15^NO${}_{3}^{-}$. Bars indicates SE for *n* = 6 (adapted from Filleur et al., 2001). **(C)** Monophasic isotherm interpretation for ^86^Rb^+^ uptake of roots from 4-5-d-old *Arabidopsis* intact seedlings (adapted from Polley and Hopkins, 1979). **(D)** Multiphasis or discontinuous isotherms interpretation for ^86^Rb^4^ uptake of excised roots from 6-d-old dark grown maize roots (log_10_*v* vs. log_10_ [Rb^+^]_ext_) (from Nissen, 1989). K^+^ influx rate was measured after 10 or 30 min of labeling at 23°C with a nutrient solution of 0.2 mM CaSO_4_ and 1 mM MES Buffer pH 6.5 containing between 0.005 and 50mM ^86^Rb^4^ (Kochian and Lucas, 1982). Arrows indicates transition for potassium uptake. **(E)** Allosteric regulation of ^86^Rb^+^ influx rate by internal K^+^ concentrating 6-d-old intact barley roots. K^+^ influx rate was measured after 10 min of labeling at 30° C with a nutrient solution of 0.5 mM CaSO_4_ containing between 0.02 and 0.16 mM ^86^Rb^+^(from Glass, 1976).

When influx of substrate j (J_j_) has been plotted against S_j_ concentrations in external solution (noted c${}_{\text{j}}^{\text{e}}$), a wide variety of curves can frequently be fitted to experimental data points: (i) curve with one arch, (ii) curve with two arches, or (iii) curve with one arch followed by a quasi-linear response, (iv) curves with more than two arches, and (v) sigmoid curves (Figure 4). For example in erythrocytes, sodium uptake between 0 and 150 mM shows a sigmoid rather than a curvilinear relationship (Garrahan and Glynn, 1967). Similarly, depending on the internal concentration of K^+^, root influx of K^+^ showed an allosteric regulation (Glass, 1976).

### Carrier viewpoint of enzyme-like modeling

The idea of modeling arches given by the experimental points obtained with unidirectional flux of tracer originated from the pioneering work of Emmanuel Epstein and his group (Epstein and Hagen, 1952; Epstein, 1953, 1966). The interpretation of ion influx isotherms is based on applying analogical reasoning to enzyme functioning (Briskin, 1995; Jacoby, 1995). In brief, this reasoning states that the absorption mechanism of a substrate S_j_ from external to internal is catalyzed by a carrier C. In this case,

(1) $$\begin{array}{l} {\text{S}}_{\text{j}}^{\text{e}}+\text{C}↔{\text{CS}}_{\text{j}}\to {\text{S}}_{\text{j}}^{\text{i}}+\text{C}, \end{array}$$

where S${}_{\text{j}}^{\text{e}}$ and S${}_{\text{j}}^{\text{i}}$ represent substrate S_j_ present in external and internal solution, respectively. CS_j_ represents the complex formed between S_j_ and the carrier. This formalism is equivalent to Michaelis–Menten kinetics formalizing an enzymatic transformation of S into P through E activation:

(2) $$\begin{array}{l} \text{S}+\text{E}↔\text{ES}\to \text{P}+\text{E}, \end{array}$$

where ES represents the enzyme-substrate complex.

The graphs {c${}_{\text{j}}^{\text{e}}$, J${}_{\text{j}}^{\text{ei}}$(c${}_{\text{j}}^{\text{e}}$)} obtained are curves with one arch (Figure 2A), and are modeled by a hyperbola of the Michaelis–Menten type stated to represent the involvement of a single carrier (Cornish-Bowden et al., 2004).

(3) $$\begin{array}{l} \text{v}=V\text{max}\cdot \left[c_{s}\right]∕\left(Km+\left[c_{s}\right]\right), \end{array}$$

where v is the velocity or “*flow,*” c_*s*_ is the external substrate concentration, *Vmax* (maximum velocity) represents the velocity of enzyme saturated by the substrate, and 1/K_m_ is an approximation of the enzyme affinity for the substrate. This equation can also be written:

(4) $$\begin{array}{l} {\text{J}}_{\text{j}}^{\text{ei}}\left({\text{c}}_{\text{j}}^{\text{e}}\right)=V\text{max}\cdot \left[c_{j}^{e}\right]∕\left(Km+\left[c_{j}^{e}\right]\right), \end{array}$$

where *Vmax* is the saturation velocity of carrier C by substrate S_j_, and 1/*Km* is an approximation of the affinity of carrier C for substrate S_j_.

For the graphs with two arches, we consider that each of the arches reflects the activity of a particular type of carrier considered to play the dominant role in the range of concentrations over which the arch is observed (Figure 4A). Each of the arches is then modeled by a Michaelis–Menten hyperbola and characterized by the values of the microscopic parameters *Vmax* and *Km* (*Vm1, Km1* for the first hyperbola and *Vm2, Km2* for the second, and so on). Because graphs with two arches are those most frequently obtained, it was widely assumed that two types of carriers or mechanisms were most often involved in the absorption process: a low maximum velocity (and so capacity) and HATS (mechanism I, called HATS for High Affinity Transport System) and a high maximum velocity, LATS (mechanism II, called LATS for Low Affinity Transport System).

When the second mechanism shows a linear part (Figure 4B), it is considered that the diffusion becomes dominant in the corresponding concentration range (Kochian and Lucas, 1982; Briskin, 1995; Jacoby, 1995; Britto and Kronzucker, 2008). When the graphs {c${}_{\text{j}}^{\text{e}}$, J${}_{\text{j}}^{\text{ei}}$(c${}_{\text{j}}^{\text{e}}$)} are sigmoid (Figure 4E), it is inferred that the corresponding carriers could be allosteric proteins (Glass, 1976).

In summary, *enzyme-like* modeling seems powerful since it determines the molecular characteristics of the carrier from the macroscopic unidirectional influx measurements of root biological samples (e.g., number of different types of carriers involved, estimated values of *Km* and *Vmax* of these carriers, possible allosteric nature of carriers). However, at the same time, a broad diversity of equations fitting experimental data points is controversial because no unity in the identification of root transporter and associated parameters is allowed.

### Discussion of enzyme-like modeling

Theoretical studies based on the modeling of realistic mechanisms for carrier functioning showed, with the help of some simplifying assumptions, that such systems could actually generate pulses obeying the Michaelis–Menten equation (King and Altman, 1956; Schachter, 1972; Wong and Hanes, 1973). However, although the arches of the experimental graphs can be reasonably modeled by a Michaelian hyperbola, this does not mean that hyperbolic Michaelis–Menten fitting is the best of all possible models for these arches made from experimental data points: in other words *enzyme-like* modeling may possibly be satisfactory, but is not necessarily so. Indeed, there is no particular reason plant roots should behave like an enzyme.

#### Molecular analyses of ion carrier mutants are inconsistent with the predictions of enzyme-like modeling

In the last two decades the cloning and molecular characterization of new macronutrient carriers such as potassium and nitrate, operating over a wide range of external concentrations, has thrived (Touraine et al., 2001; Britto and Kronzucker, 2008; Wang et al., 2012). The mutant analyses validated the existence of complex carrier systems for root absorption rather than a simple carrier system over low and high ranges of potassium and nitrate concentrations (Alemán et al., 2011; Le Deunff and Malagoli, 2014b; Krapp, 2015). For example, the dual affinities of some K^+^ and NO${}_{3}^{-}$ transporters as a result of protein modifications such as phosphorylation and dephosphorylation invalidate the notion of distinct high and low affinity transport systems established by the *enzyme-like* approach (Liu and Tsay, 2003; Cheong et al., 2007; Ho et al., 2009; Ragel et al., 2015). It also denies the oversimplification of carrier insertion in one single membrane (Crawford and Glass, 1998; Le Deunff and Malagoli, 2014b). In addition, the redundancy of the genes encoding nitrate transporters in *Arabidopsis* operating in a low range of external concentrations (< 1 mM) such as NRT2.1, NRT2.2, NRT2.4, NRT2.5, and NRT1.1 (NPF6.3) also invalidates *enzyme-substrate* analogical reasoning (Li et al., 2007; Kiba et al., 2012; Glass and Kotur, 2013; Kotur and Glass, 2014; Lezhneva et al., 2014). Furthermore, the recent discovery of new gene families of nitrate transporters: *CLC* (ChLoride Channel) and *NAXT* (NitrAte eXcretion Transporter) has increased the complexity of the root catalytic device for nitrate (De Angeli et al., 2006; Segonzac et al., 2007). The ClCa transporter is involved in nitrate influx into the vacuole and participates in the short-distance transport of nitrate and the homeostasis for cellular nitrate (Monachello et al., 2009; Krebs et al., 2010). Likewise, impairment of nitrate vacuolar sequestration in a mutant defective in tonoplast proton pump, or inhibition of the proton pump using pharmacological inhibitors, up-regulated the *AtNRT1.5* gene expression and down-regulated *AtNRT1.8* expression (Han et al., 2016). The NRT1.5 nitrate transporter is involved in nitrate xylem loading, while the NRT1.8 gene is responsible for xylem unloading (Lin et al., 2008; Li et al., 2010; Chen et al., 2012; Zhang et al., 2014). These results demonstrate that the regulation of the cytosolic nitrate concentration in roots regulates the long-distance transport of nitrate from roots to shoots and the nitrate influx at the root plasma membrane (Geelen et al., 2000; Monachello et al., 2009). They corroborate the previous conclusion of compartmental analysis by the tracer efflux method showing that influx of nitrate to roots is highly regulated by nitrate import into the vacuole, efflux from the cell and loading into the xylem (Pitman, 1977; Britto and Kronzucker, 2001b, 2003a,b). This molecular complexity will certainly go on increasing with the identification of other genes encoding nitrate carriers involved in nitrate influx and efflux from the vacuole (Migocka et al., 2013) or nitrate xylem loading and unloading (Köhler et al., 2002; Han et al., 2016).

Hence the overall root organ should be considered as a catalytic device across the root radius, formed by a complex of nitrate transporters (CNT) operating at low and high ranges of external concentrations (Tinker and Nye, 2000; Britto and Kronzucker, 2003a). The compartment location and inducibility of nitrate transporters conflicts with the implicit interpretation of *enzyme-substrate* modeling where *Vmax* and *Km* are constant parameters and where nitrate transporters are located in a “single root membrane.” This probably explains the varied shapes of isotherms encountered in the literature under the different experimental conditions used (Figure 4).

#### Macroscopic vs. microscopic parameters

The above arguments do not completely invalidate the *enzyme-like* modeling approach. It is easier to manipulate the macroscopic values taken by a few parameters (*V*${}_{m}^{app}$ and *K*${}_{m}^{app}$, apparent maximum velocity and affinity constant) than to manipulate the experimental values or even the plot that can be drawn from these values. However, we must face the fact that *V*${}_{m}^{app}$ and *K*${}_{m}^{app}$ do not have the molecular meanings we might expect (*Vm* and *Km* from an enzymatic reaction). It is clear that parameters *Vmax* and *Km* are only “apparent” parameters, i.e., they reflect activity of ion uptake at the root level and the subsumed activity of several elemental transporters (Neame and Richards, 1972; Polley and Hopkins, 1979; Briskin, 1995; Tinker and Nye, 2000; Franks, 2009). Unfortunately, *V*${}_{m}^{app}$ and *K*${}_{m}^{app}$ are too often regarded as actual values of microscopic parameters at the elemental transporter level (Siddiqi et al., 1989, 1990; Forde and Clarkson, 1999; Tinker and Nye, 2000). For the absorption process, *V*${}_{m}^{app}$ and *K*${}_{m}^{app}$ are only macroscopic parameters describing the overall behavior of the root sample studied for the absorption process considered in the experimental conditions used. The major difficulty in using these macroscopic parameters arises from the fact that we are unable (i) to find a simple meaning for them in relation to the integrated constitution and functioning of the root sample at a molecular level and (ii) to fill the gap between the transporters and the unidirectional or net flux measured at root level. Some of the most serious shortcomings of the *enzyme-substrate* interpretation to describe nutrient ion uptake have been corrected in the ecological models of phytoplankton in the last three decades (see Section: Changes in the Number and Nature of Transporters Involved in Nutrient Uptake Modify *Vmax* and *Km* values and Section: Inducibility of Nutrient Transporters in Relation with Plant Nutrient Status also Modifies Apparent Values of *Vmax* and *Km* below).

## Flow-force modeling

### Stating the problem

Non-equilibrium thermodynamics may be a useful frame for a macroscopic description of the substrate-absorption in which the parameters have a straightforward meaning with respect to the biological sample studied (Katchalsky and Curran, 1965; Thellier et al., 2009; Thellier, 2012).

Briefly, let us consider a system, the “internal” and “external” compartments of which are termed “i” and “e,” respectively. The system may be defined by state variables that are either intensive (temperature [in K], pressure, electric potential, chemical potential of a substance, etc.) or extensive (entropy, volume, quantity of electricity, quantity of a substance, etc.). Intensive and extensive variables can be coupled or “conjugated”: temperature/entropy, pressure/volume, electric potential/quantity of electricity, chemical potential of a substance/quantity of that substance. Generally speaking, the properties of extensive variables are such that they cannot be defined at a point but only in macroscopic systems or subsystems (e.g., the volume of a point is meaningless) and they are additive (e.g., with a system made up of subsystems, the content of a substance in the system is the sum of the contents of that substance in the subsystems). By contrast, the properties of intensive variables are defined at a mathematical point and are not additive. For instance, when we speak about “the temperature of a system,” we imply that all the points in the system are at that temperature. If one or several state variables of a system do not keep the same value in time, this system is said to undergo a “transformation” (or “process”). Two different types of transformation may occur: (i) an exchange of an extensive variable between the internal, i, and the external, e, compartments of the system and/or (ii) a chemical reaction within the system. Let us consider the case of an exchange. The exchange is driven by forces resulting from potential gradients. In the simple case (that considered here) of an exchange of an extensive variable such as a chemical potential of a substance (S_j_) between i and e through an infinitely thin frontier, the driving force is merely the difference in the value of the conjugate intensive variable in e and i. In the case presented, this is the difference in the concentration of substance J (namely, c${}_{\text{j}}^{\text{i}}$ and c${}_{\text{j}}^{\text{e}}$). The “*flow,*” J_j_, of S_j_ between e and i is the quantity of S_j_ exchanged per unit of time. Using isotopic tracers, it is easy to measure the influx, J${}_{\text{j}}^{\text{ei}}$, and the efflux, J${}_{\text{j}}^{\text{ie}}$, of S_j_ separately with:

(5) $$\begin{array}{l} {\text{J}}_{\text{j}}={\text{J}}_{\text{j}}^{\text{ei}}-{\text{J}}_{\text{j}}^{\text{ie}} \end{array}$$

When close enough to equilibrium (i.e., when c${}_{\text{j}}^{\text{i}}$ and c${}_{\text{j}}^{\text{e}}$ no longer fluctuate in the case of a substance), the *flow*, J_j_, is a linear function of the *force*, X_j_ (i.e., C_j_ in the case of a substance):

(6) $$\begin{array}{l} {\text{J}}_{\text{j}}={\text{L}}_{\text{j}}\cdot {\text{X}}_{\text{j}}, \end{array}$$

in which the coefficient L_j_ is termed the “conductance” of the process. Farther away from equilibrium, the relation between *flow* and *force* becomes non-linear (Thellier, 1973).

### Application of flow-force relationships to the transport process

Transposing Equation (1) for a transport process in cell systems in which i is the cytosol of the root cells and e is the apoplastic spaces results in the net *flow* of a substance S_**j**_:

(7) $$\begin{array}{l} {\text{J}}_{\text{j}}\left({\text{c}}_{\text{j}}^{\text{e}}\right)={\text{J}}_{\text{j}}^{\text{ei}}\left({\text{c}}_{\text{j}}^{\text{e}}\right)-{\text{J}}_{\text{j}}^{\text{ie}}\left({\text{c}}_{\text{j}}^{\text{e}}\right), \end{array}$$

where J_j_(c${}_{\text{j}}^{\text{e}}$) is the positive *flow* (influx) from e to i and J${}_{\text{j}}^{\text{ie}}$(c${}_{\text{j}}^{\text{e}}$) is the positive *flow* (efflux) from i to e. Based on the general *flow-force* theory as set out above, we may consider that in a biological system (here a root cell) in the presence of two substances S_j_ and S_k_, the *flow* of S_j_ depends not only on “combined” terms (i.e., difference in concentrations across the cell membrane), but also on “crossed” terms (i.e., the effect of S_k_ on flow of S_j_, for instance). It is said that there is a “coupling” between these two processes. Two well-known examples of couplings are: (i) the coupling between the transport process of a substance, S_j_, and a reaction process, R (active transport of first order) and (ii) the coupling of the transport process of a substance, S_j_, to the transport process of another substance, S_k_ (active transport of the second order, Mitchell, 1967; Hanson, 1978). *Flow-force* modeling may be proposed to simulate ion *flows* across the root membrane based on driving forces, and not only the putative enzymatic deduced functioning of carriers (Thellier et al., 2009; Thellier, 2012). To deal with linear equations, the transport process, as governed by differences in substrate concentrations, has to be close enough to equilibrium. If °c${}_{\text{j}}^{\text{e}}$ is the concentration of the growth medium with which plants have pre-equilibrated, the experiments will have to be carried out using a series of external concentrations of S_j_, c${}_{\text{j}}^{\text{e}}$, close enough to °c${}_{\text{j}}^{\text{e}}$. Accordingly, washing the roots before the absorption experiments (as is commonly reported in numerous influx measurement experiments) would strongly disturb the pre-equilibration value, and so ultimately the thermodynamic conditions and *forces* driving *flow*. Washing (with calcium salt, for instance) before influx measurement should therefore be avoided.

### The optimal experimental protocol for flow-force modeling

The experimental protocol (Thellier et al., 2009) best suited to application of the *flow-force* approach is as follows. We prepare a series of growth vessels containing a nutrient solution with the initial concentration (°c${}_{\text{j}}^{\text{e}}$) of S_j_. We dip plant samples in these growth vessels long enough for them to equilibrate with this medium with regard to S_j_. Without removing the plant samples from the vessels, we impose various concentrations, c${}_{\text{j}}^{\text{e}}$, of S_j_ in the growth vessels by adding either small amounts of S_j_ (c${}_{\text{j}}^{\text{e}}$ > °c${}_{\text{j}}^{\text{e}}$) or small volumes of a solution identical to the initial medium except that it contains no S_j_ (c${}_{\text{j}}^{\text{e}}$ < °c${}_{\text{j}}^{\text{e}}$). This enables us to smoothly increase or decrease test concentration of substance J. For each value of c${}_{\text{j}}^{\text{e}}$ thereby obtained, influx, J${}_{\text{j}}^{\text{ie}}$ (c${}_{\text{j}}^{\text{e}}$), and efflux, J${}_{\text{j}}^{\text{ei}}$ (c${}_{\text{j}}^{\text{i}}$) of S_j_ can be measured using labeled medium marked with a suitable isotope with unlabeled plant samples (direct influx measurements) or unlabeled medium with pre-labeled plant samples (efflux measurements). Accordingly, the net flux for each value of c${}_{\text{j}}^{\text{e}}$, J_j_(c${}_{\text{j}}^{\text{e}}$) can be obtained easily using Equation (3).

### The flow-force model

Using reasonable simplifying assumptions such as (i) similar values for the activity coefficient of the substrate in the internal and external media, (ii) quasi-constant transmembrane electrical potential over the range of values of c${}_{\text{j}}^{\text{e}}$, and (iii) constant export of the substrate S_j_ to the aerial parts over the duration of the experiments, the following equation expressing *flow* of substance J across a membrane can be written:

(8) $${\text{J}}_{\text{j}}({\text{c}}_{\text{j}}^{\text{e}})=\text{RT}\lambda_{\text{j}}\text{ln}(({\text{c}}_{\text{j}}^{\text{e}})/{(}^{{}^{\circ}}{\text{c}}_{\text{j}}^{\text{e}}))={\text{L}}_{\text{j}}\text{ln}(({\text{c}}_{\text{j}}^{\text{e}})/{(}^{{}^{\circ}}{\text{c}}_{\text{j}}^{\text{e}})),$$

with

(9) $$\begin{array}{l} {\text{L}}_{\text{j}}=\text{RT}\lambda_{\text{j},} \end{array}$$

where R is the gas constant, T the absolute temperature and λ_j_, the overall conductance of the sample for the net uptake of S_j_. Table 1 presents the parameters of models, their symbols and their units. When a change in the experimental conditions causes a change λ_j_, an Arrhenius diagram lets us determine whether the change is quantitative or qualitative (see Thellier et al., 2009 for further explanations).

**Table 1:** **Key to symbols used in the text**.

**Symbol**	**Description**	**Unit**
*e*	Apoplastic space of the root cells	
*i*	Internal space of the root cells	
${}^{{}^{\circ}}c_{j}^{e}$	Initial concentration in the bulk solution	mol. m^−3^
$c_{j}^{i}$	Concentration of Sj component in the cytosol	mol. m^−3^
$c_{j}^{e}$	Concentration of Sj component in the apoplast	mol. m^−3^
*j*	Solute under study	
*J*j**	Solute influx (based on membrane area)	mol. m^2^. s^–1^
$K_{m}^{app}$	Apparent half saturation constant	mol. m^−3^
*Km*	Half saturation Michaelis-Menten constant	mol. m^−3^
*L*j**	Conductance of the overall root system	mol. h^−1^.g^−1^ root DW
*Ln*	Logarithm to base e	
*π*j**	Effect of all the processes energizing the transport of Sj	mol^−1^.m^3^
*R*	Gas constant	8.314 J mol^−1^ K^−1^
*S*j**	Solute concentration of solute J in the bulk solution	mol. m^−3^
*T*	Absolute temperature	K or °C
$V_{m}^{app}$	Apparent maximal uptake rate	mol. s^−1^
*Vmax*	Maximum reaction velocity of Michaelis-Menten	mol. s^−1^

A more general expression of net *flows* that can be used when the simplifying assumptions are not properly fulfilled would be:

(10) $$\begin{array}{l} {\text{J}}_{\text{j}}\left({\text{c}}_{\text{j}}^{\text{e}}\right)={\text{L}}_{\text{j}}\text{ln}\left(\pi_{\text{j}}\cdot \left({\text{c}}_{\text{j}}^{\text{e}}\right)\right), \end{array}$$

where π_j_ characterizes the resulting effect of all terms (i.e., activity coefficients, electric potential difference, potential couplings, etc.) other than c${}_{\text{j}}^{\text{e}}$ involved in the driving force for the net absorption of S_j_ by the plant sample under study. This means that when a system of semi-log coordinates is used {ln (c${}_{\text{j}}^{\text{e}}$), J_j_(c${}_{\text{j}}^{\text{e}}$)}, the plot representing the experimental points is expected to be quasi-linear for the values of c${}_{\text{j}}^{\text{e}}$ sufficiently close to the equilibrium concentration °c${}_{\text{j}}^{\text{e}}$ (Thellier et al., 2009). Hence it is expected that if the experiment is undertaken under optimal conditions (similar values of activities coefficients, no change in electrical potentials across the membrane and constant value of net flux across tonoplast and up to aerial parts), the intersection of plots on the abscissa (i.e., *flow* = 0) estimates π_j_ as:

(11) $$\begin{array}{l} ln\left(1{∕}^{{}^{\circ}}c_{\text{j}}^{\text{e}}\right)=ln\pi_{\text{j}.} \end{array}$$

**Figure 5:**
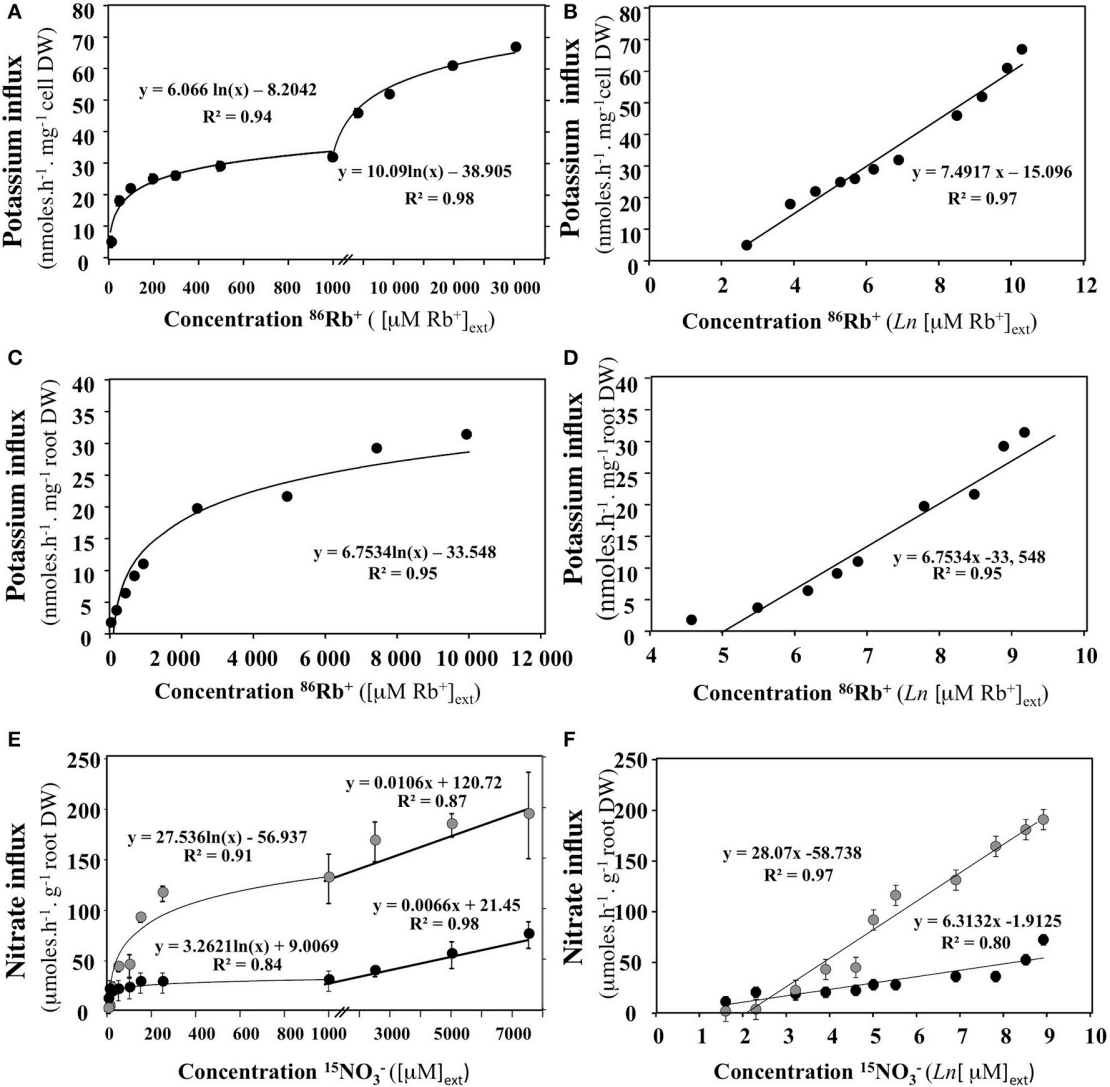
**Transformation of nitrate or potassium uptake rate isotherms in semi-log coordinates {ln (c${}_{\text{j}}^{\text{e}}$), J_j_(c${}_{\text{j}}^{\text{e}}$)}**. **(A,B)** Rate of potassium (^86^Rb^+^) uptake by cells of *chlorella pyrenoidosa* as a function of and transformation of the data in semi-log coordinates (From Kannan, 1971). Verticla bars represent ±SD of the means. **(C,D)** Rate of Potassium (^86^Rb^+^) uptake by roots of *Arabidpopsis* intact seedlings as a function of KCI concentration in the medium and transformation of the data in semi-log coordinates (from Polley and Hopkins, 1979). **(E,F)** Rate of nitrate uptake by roots of *Brassica napus* intact seedlings as a function of KNO3 concentration in the medium and transformation of the data in semi-log coordinates (from Faure-Rabasse et al., 2002). Plants were either non-induced (grown without NO3 supply, black circles) or induced during 24 h by 1 mM KNO3 prior to measurements (gray circles). Vertical bars indicated ±SD for *N* = 3 when larger than the symbol.

Conversely, if the experimental protocol is not optimal, then the plot intersects the abscissa again at a point −ln π_j_, but where π_j_ is no longer equal to 1/°c${}_{\text{j}}^{\text{e}}$, although it remains the result of the contribution of all terms other than c${}_{\text{j}}^{\text{e}}$ involved in the driving forces energizing the absorption of S_j_. In such a case, difference in activity coefficient, transmembrane potentials and fluxes into cells may all be single or combined candidates contributing in a significant way to *forces* driving *flow*. This underlines the importance of conducting experiments close to equilibrium in order to avoid confounding effects when investigating and ultimately modeling *flows*. In other words, −ln π_j_, represents a thermodynamic constant that accounts for energy coupling necessary for Sj transport. The plot in Figure 5 illustrates some examples of representations in semi-log coordinates {ln (c${}_{\text{j}}^{\text{e}}$), J_j_(c${}_{\text{j}}^{\text{e}}$)} for potassium and nitrate uptake from data points obtained in intact plants and unicellular algae in the literature (Kannan, 1971; Polley and Hopkins, 1979; Faure-Rabasse et al., 2002).

### Quantitative/qualitative modifications of the global conductance, λ_j_, in response to a change in the experimental conditions

**Figure 6:**
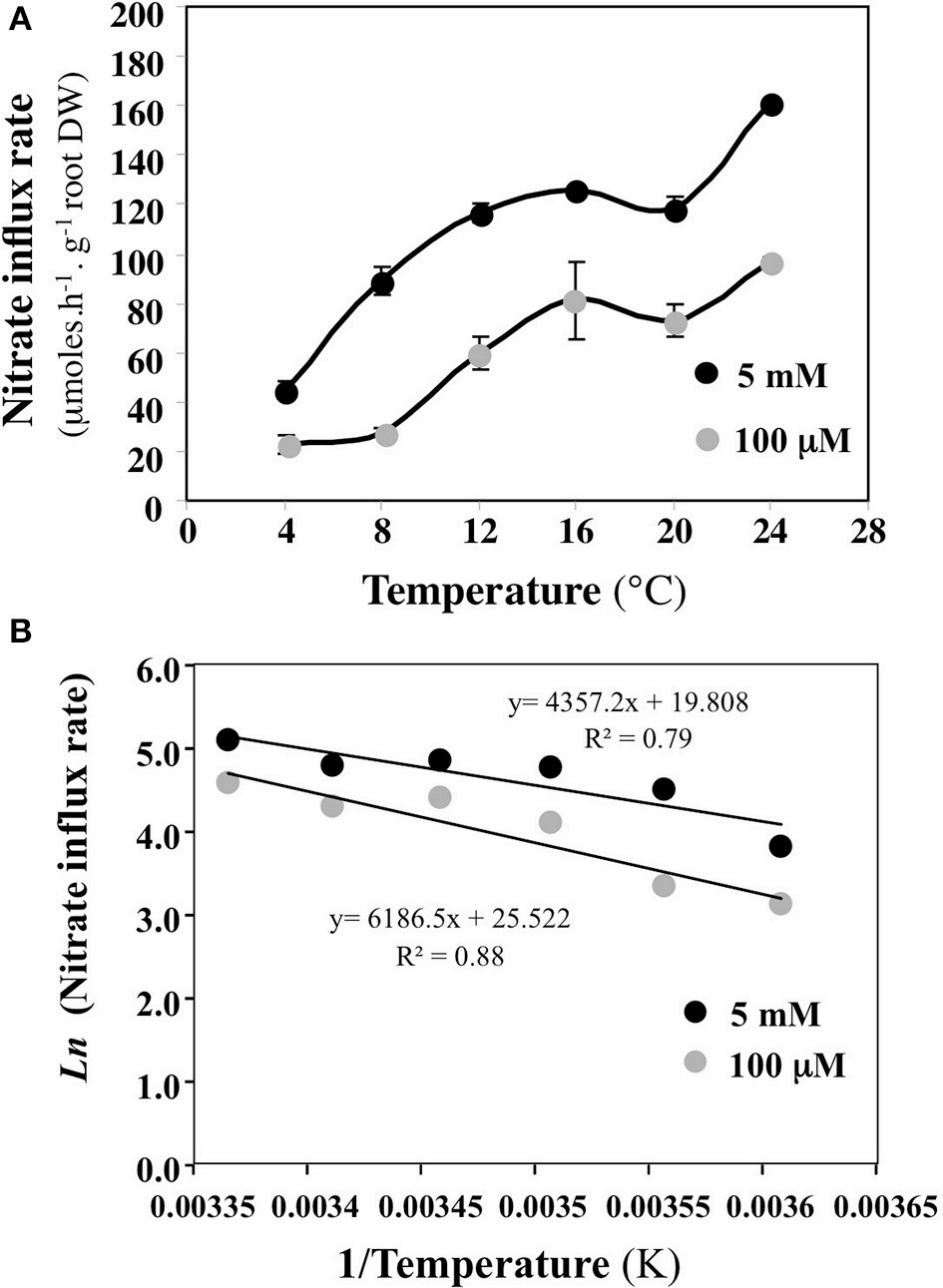
**Building an Arrhenius plot from experimental data points to check qualitative or quantitative modifications or the root catalytic device for nitrate uptake. (A)** Variations of nitrate uptake rate at 100 μM and 5 mM external nitrate concentration induced by different root temperature treatments. **(B)** Arrhenius diagrams {1/T, log J_j_) deduced from nitrate influx rate variations in response to root temperature changes. Vertical bars indicated ±SD for *N* = 3 when larger than the symbol.

When a change in experimental conditions (e.g., use of younger or older plants) causes a change of λ_j_, this change can come from quantitative modification of the root catalytic device (which gathers all transporters) involved in the absorption of S_j_ (change in the number of molecules of carriers) and/or qualitative changes (change in the nature or activity of the carriers). In the range of the biological temperatures [i.e., between 275 (1.85°C) and 305 K (31.85°C)], different values of λ_j_ are obtained for different temperature values (all the other variables unchanged). Using an Arrhenius plot {1/T, log J_j_} or {1/T, λ_j_}, the experimental points are expected to lie on a straight line, the slope of which plays a role comparable to that of an activation energy for the overall process of absorption under consideration (Thellier, 1971). When the experiments are carried out with two different conditions (for instance using young or adult plants or at two different external concentrations S_j_), if the two plots thus obtained are parallel to each other (similar slope), then the overall absorption processes differ quantitatively (e.g.*,* the density of carriers at the root epidermis is not the same). By contrast, if the plot slopes are significantly different, then a qualitative change has occurred (e.g., in the specific activity of the carriers). Figure 6 illustrates the Arrhenius diagram obtained by plotting the logarithm of nitrate influx J_j_ vs. 1/root temperature at 100 μM and 5 mM external nitrate concentration (Le Deunff and Malagoli, 2014a). Although the experiment was not carried out in the best conditions, the parallel behavior of the two linear curves (Figure 6B) highlights that the temperature does not change the root conductance for nitrate at 100 μM and 5 mM. Hence the increase in nitrate influx with temperature is not associated with changes in the catalytic efficacy of the root catalytic device for nitrate (specific activity of carriers), but is instead associated with quantitative changes such as the numbers of nitrate carriers.

### Application of flow-force modeling under suboptimal experimental conditions

To our knowledge, no experiment has ever been carried out under the optimal conditions indicated in Section: Application of Flow-Force Relationships to the Transport Process and Section: The Optimal Experimental Protocol for Flow-Force Modeling. However, making a few simplifying hypotheses (in particular the assumption that the efflux remains small compared with the influx over the range of concentrations used, which means that the net *flow* is not too different from the influx), it is possible to apply *flow-force* modeling to the numerous experiments carried out since isotopic tracers became available (Thellier et al., 2009; Le Deunff and Malagoli, 2014a).

### Initial approach to the problem: the electrokinetic formalism

*Flow-force* modeling of cell transports was initially introduced using the “electrokinetic formulation,” based on a formal analogy with classical electrokinetics (Thellier, 1969, 1970a,b, 1973). This does not change the main equations given above. The reason is that classical electrokinetics amounts to *flow-force* modeling in the linear domain.

## Attempts to correct the shortcomings of enzyme-substrate interpretation in the nutrient uptake models

For over 50 years, the *enzyme-substrate* interpretation of isotherm kinetics has prevailed, and has been extended to models of nutrient ion uptake in plants (Barber, 1995; Le Bot et al., 1998; Tinker and Nye, 2000; Ma et al., 2008) and phytoplankton (Dugdale, 1967; MacIsaac and Dugdale, 1969; Morel, 1987; Smith et al., 2009, 2014; Aksnes and Cao, 2011; Fiksen et al., 2013). Among all the models, the phytoplankton ecological models of nutrient uptake based on the Michaelis–Menten (MM) formalism show the most noteworthy developments for coping with deviations between simulated and measured outputs. The development of these phytoplankton ecological models encapsulates all the problems encountered by the rigid values of *Vmax* and *Km* provided by the MM models developed in plants (Aksnes and Egge, 1991; Franks, 2009).

### Changes in the number and nature of transporters involved in nutrient uptake modify *Vmax* and *Km* values

Phytoplankton physiologists have found that one of the main problems of MM models is the rigid values of the *Vmax* and *Km* obtained in short-term experiments in oligotrophic and eutrophic regimes (Aksnes and Egge, 1991; Franks, 2009; Bonachela et al., 2011; Fiksen et al., 2013; Smith et al., 2014). Plant physiologists reached the same conclusion in the 1970s (Nye and Tinker, 1969; Jungk et al., 1990; Barber, 1995; Tinker and Nye, 2000). Several experiments have demonstrated the shortcomings of MM kinetics to describe nutrient ion uptake of phytoplankton (Droop, 1970, 1973; Aksnes and Egge, 1991; Franks, 2009). The observed co-variation of *Vmax* and *Km* values has invalidated the definition of *Km* as an affinity constant, and demonstrated that the *Vmax* and *Km* are only apparent parameters (*V*${}_{m}^{app}$ and *K*${}_{m}^{app}$) and cannot be regarded as constant values depending for instance on time or internal nutrient status (Neame and Richards, 1972; Aksnes and Egge, 1991; Franks, 2009; Aksnes and Cao, 2011). Hence recent trait-based phytoplankton models provide novel mechanistic expressions to tackle and correct the static expression of the *enzyme-substrate* formalism (Aksnes and Egge, 1991; Aksnes and Cao, 2011; Bonachela et al., 2011; Fiksen et al., 2013; Smith et al., 2014). By deriving equation of nutrient transport at cellular level rather than at enzymatic individual level, Aksnes and Egge (1991) demonstrate that MM modeling is a special case of their mechanistic model. In these models, the uptake of nutrient ions depends on the number and density of uptake sites on the plasmalemma of phytoplankton cells, which determines the plasticity of the uptake apparatus in response to temperature and nutrient diffusion in relation with nutrient regimes (Aksnes and Egge, 1991; Fiksen et al., 2013; Lindemann et al., 2016). In addition, the affinity constant α, defined as the Vmax/Km ratio, is preferred to Km because it represents the area of the cell membrane able to catch nutrient ions and it is proportional to the cell size. The introduction of mechanistic parameters in these extended MM models avoids bias up to 50% in some configurations compared with usual MM models (Fiksen et al., 2013), showing the pertinence of the approach.

### Inducibility of nutrient transporters in relation with plant nutrient status also modifies apparent values of *Vmax* and *Km*

In plants, activities of nutrient transporters such as NO${}_{3}^{-}$, PO${}_{4}^{2-}$, K^+^, and SO${}_{4}^{2-}$ are modified by external nutrient availability and pre-treatment with the nutrient under study, which alter nutrient status in plants (Glass, 1976; Siddiqi et al., 1989, 1990; Jungk et al., 1990). Thus in *Arabidopsis*, it has been clearly demonstrated that *AtNRT2.1, AtNRT2.2,* and *AtNRT1.1* nitrate transporter genes are induced by external nitrate (Tsay et al., 1993; Amarasinghe et al., 1998; Krapp et al., 1998). Transcriptional induction depends on plant N status, the level induction decreasing with increasing nitrate concentration during the pre-treatment (Siddiqi et al., 1989, 1990). After the induction, a steady de-induction process is observed, with a reversion after 48–72 h to the initial value of nitrate influx rate before induction (Faure-Rabasse et al., 2002; Okamoto et al., 2003). These results demonstrate that depending on plant N status and nitrate pre-treatment, values for the parameters *Vmax* and *Km* can be determined, but are not constant. Although in phytoplankton, inducible behavior of some nutrient carriers such as nitrate transporters has been recently discovered (Rogato et al., 2015), nutrient uptake regulation by N status was already taken into account in trait-based models through a modulating term dependent on the internal nutrient concentration or N/C ratio (Droop, 1970, 1973; Geider et al., 1998; Litchman et al., 2007; Litchman and Klausmeier, 2009; Bonachela et al., 2011).

Despite the quantitative use of the *enzyme-substrate* approach in nutrient uptake models (N, P, K, S) in plants and phytoplankton, the thermodynamic processes involved in nutrient ion uptake and the realistic solutions offered by the *flow-force* modeling approach can no longer be ignored. One of the most severe limitations of MM models in plants and phytoplankton is that temperature, which partly drives biochemical reaction rates and ion diffusion processes, and which in turn modify parameters *Vmax* and *Km*, is not taken into account (Aksnes and Egge, 1991; Tinker and Nye, 2000; Fiksen et al., 2013). Nutrient ion kinetics are established under isothermal conditions. Response of the uptake process to temperature is thus left out of the MM model, even though temperature is a key variable acting either directly (on carrier functioning) or on nutrient availability in the diffusion boundary layer around phytoplankton cells or roots (Aksnes and Egge, 1991; Smith, 2011; Fiksen et al., 2013). The temperature dependence of nutrient influx rate in plants is well illustrated in Figure 6 for nitrate. Use of *flow-force* formalism for nutrient uptake, which includes the temperature dependence of the uptake, might greatly improve plant and phytoplankton models in response to changes in environmental variables (e.g., temperature, nutritional regimes). Likewise, the nitrate pretreatment with 1 mM KNO_3_ for 24 h on previously starved *B. napus* plants induced contrasting root conductance for nitrate and so different catalytic efficiencies (Figures 5E,F). Therefore, the embedding in *flow-force* models of the mechanistic approach used in the trait-based approach developed in phytoplankton models (cell size, number of uptake sites per cell, uptake site handling time, affinity of a single uptake site, etc.) should further improve their formalisms and enhance their performance (Lindemann et al., 2016). Attempts at flexible approaches to the uptake parameters in nutrient uptake models such as the introduction of mechanistic support for the uptake sites or cross-combination of the *flow-force* formalism with *in planta* and environmental factor effects, predicts higher and more realistic nutrient uptake rates than the usual MM counterparts (Bonachela et al., 2011; Fiksen et al., 2013; Le Deunff and Malagoli, 2014a; Malagoli and Le Deunff, 2014). Outputs of these new conceptual models demonstrate that usual nutrient uptake models based on the *enzyme-substrate* formalism are inevitably forced by some parameters to match measured nutrient taken up (Ma et al., 2008; Franks, 2009). Unlike phytoplankton nutrient uptake models, the nutrient uptake models in plants also have to allow for the effects of the growth, geometry, and aging of the root system that affect the nutrient uptake. The next sections explain how these effects are taken into account in recent modeling approaches.

### Flow-force agronomic models for nutrient uptake with one spatial dimension

Agronomic models of nutrient ion uptake in one spatial dimension (1-D models) depend on measurements of root distribution profile in the different soil layers from the soil surface to rooting depth along the growth cycle. The relationship between root development and rooting depth is generally described by an experimentally measured heuristic law that gives root distribution for different times throughout the plant growth cycle. This law accounts for the root length density distribution in one spatial dimension (Gerwitz and Page, 1974). From this framework, a new mechanistic structural-functional model for nitrate uptake was developed for a crop of winter oilseed rape (*Brassica napus* L.). The functional component of the model derives from a revisited conceptual framework that combines the thermodynamic *flow-force* interpretation of nitrate uptake isotherms and environmental and *in planta* effects on nitrate influx (Le Deunff and Malagoli, 2014a). The structural component of the model is based on estimation of root biomass contributing actively to N uptake using the determination of a synthetic parameter IRSA (Integrated Root System Age) that allows assignment of a root absorption capacity at a specific age of the root (Gao et al., 1998; Malagoli and Le Deunff, 2014). This model of one spatial dimension (1-D model) is able to respond more realistically to external nitrate fluctuations throughout the plant growth cycle under field conditions for three levels of N fertilization at both functional and structural levels (Malagoli and Le Deunff, 2014). In this model, it is assumed that convection and diffusion of nitrate ions to the root surface are optimal because the soil water content is close to field capacity. Likewise, no root competition for nitrate uptake or effects of root exudates, microbial activity and mycorrhizae are taken into account. Nitrate influx depends on fluctuation of soil nitrate concentrations, changes in climatic (temperature and PAR) and *in planta* factors (day-night and ontogenetic cycles), and changes in root uptake capacities with aging throughout the plant growth cycle (Le Deunff and Malagoli, 2014b).

### Toward flow-force agronomic models with two and three spatial dimensions

Two- and three-dimensional models for nutrient ion uptake have been developed to take into account the spatial geometry of root systems and the dynamics of water and nutrient availability and their spatial distribution in soil during the growth cycle (Somma et al., 1998; Roose, 2000; Biondini, 2008; Tournier, 2015). In general, these models were built to find analytical solutions to differential equations provided by equations of soil ion convection-diffusion and root ion influx isotherms (Roose et al., 2001; Roose and Kirk, 2009). Analytical solutions are obtained for one single cylindrical root for isothermal conditions, and then extended to the whole root system (Roose, 2000; Roose et al., 2001). Because analytical solutions can now be derived from almost any form of nutrient uptake function (Roose and Kirk, 2009), we propose to use the thermodynamic *flow-force* linear formalism of nutrient ion isotherms (Equation 10) instead of the non-linear formalism of Michaelis–Menten (Equation 4). This will make parametrization simpler to obtain a more realistic analytical expression for nutrient uptake by a single cylindrical root.

Although parameter L_j_ in Equation (9; root conductance for substrate S_j_) is taken as constant to solve the equations, it may not be truly constant. For example, the effects of fluctuating temperature throughout the growth cycle, spatial heterogeneity of nitrate in the soil, root aging, the day-night cycle and ontogenesis, which modify J_j_(c${}_{\text{j}}^{\text{e}}$) through changes in L_j_, are not taken into account in this approach (Le Deunff and Malagoli, 2014a,b). Accordingly, obtaining analytical solutions to these equations will not completely solve the problems associated with extension of the uptake behavior of one root segment to the entire root system throughout the plant growth cycle. In particular, the building of a realistic root network is confronted with the patterning process of root systems caused by the spatial heterogeneity of available water and nutrient ions in soil throughout the growth cycle (Drew, 1975; Bao et al., 2014).

Instead of finding analytical solutions and scaling up the uptake behavior of one root segment to the entire root system, mechanistic 3-D models have been developed that numerically solve nonlinear partial differential equations coupling soil water and nutrient transport with root uptake at the single root scale (Somma et al., 1998; Doussan et al., 2006; Javaux et al., 2008; Tournier et al., 2015). Thanks to recent advances in scientific computing, such models are now able to simulate water and nutrient transport with root uptake for realistic root systems, taking advantage of unstructured grids adapted to the complex geometry of the root system and solving the computationally intensive discrete problems on parallel architectures (Tournier, 2015). Like for 2-D models, the *flow-force* formalism can be easily introduced in this type of 3-D model.

## Concluding remarks

Major benefits of the *flow-force* formulation are that it makes experimentally testable predictions and it expresses the results of macroscopic measurements (i.e., made on an entire biological sample) by macroscopic parameters (L_j_ and π_j_) associated with a biological meaning, without considering molecular characteristics of carriers. It also provides a coordinate graph {ln (c${}_{\text{j}}^{\text{e}}$), J_j_(c${}_{\text{j}}^{\text{e}}$)} that is linear if c${}_{\text{j}}^{\text{e}}$ values are sufficiently close to the equilibrium concentration of S_j_ (°c${}_{\text{j}}^{\text{e}}$). The kinetic patterns can be improved by changing the pre- and post-wash procedures for roots before net influx rate measurements in order to come as close as possible to equilibrium conditions. In addition, linear formalism of the *flow-force* approach could usefully replace the Michaelis–Menten formalism of the *enzyme-substrate* approach currently used in the phytoplankton and agronomic nutrient ion uptake models (Barber, 1995; Tinker and Nye, 2000; Roose and Kirk, 2009; Tournier, 2015). This would offer a simplification of parametrization to help find more realistic analytical expressions and numerical solutions for ion uptake in 2-D and 3-D models of nutrient uptake in plants.

## Author contributions

Substantial contributions to the conception or design of the work: EL, PT, PM. Drafting the work or revising it critically for important intellectual content: EL, PT, PM. Final approval of the version to be published: EL, PT, PM. Agreement to be accountable for all aspects of the work in ensuring that questions related to the accuracy or integrity of any part of the work are appropriately investigated and resolved: EL, PT, PM.

### Conflict of interest statement

The authors declare that the research was conducted in the absence of any commercial or financial relationships that could be construed as a potential conflict of interest. The reviewer, ID, and handling Editor declared their shared affiliation, and the handling Editor states that the process nevertheless met the standards of a fair and objective review
